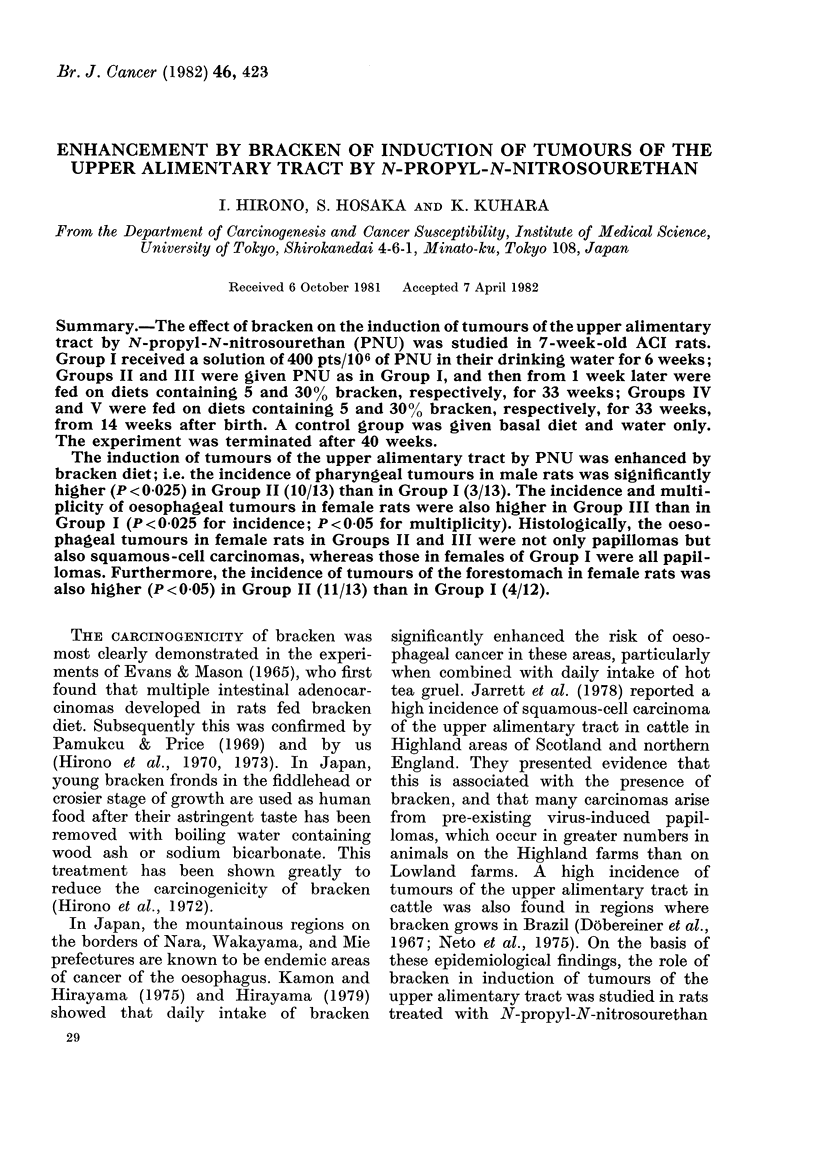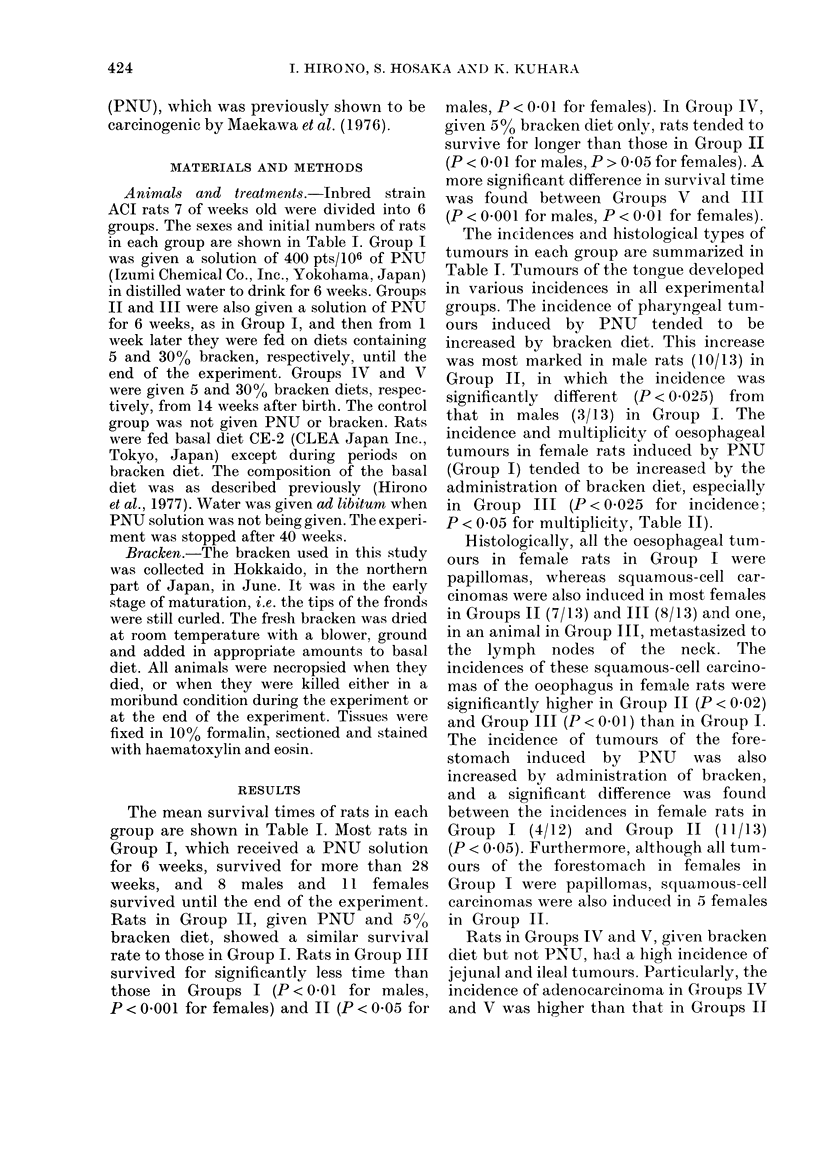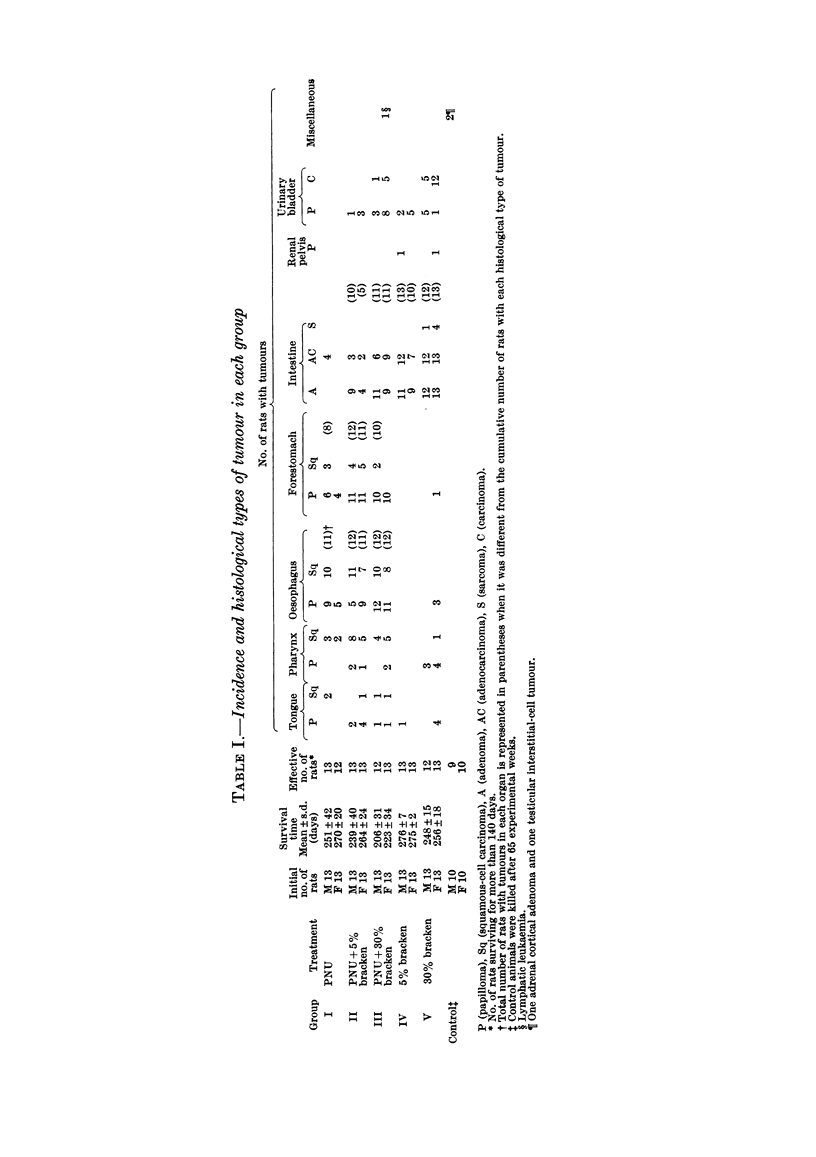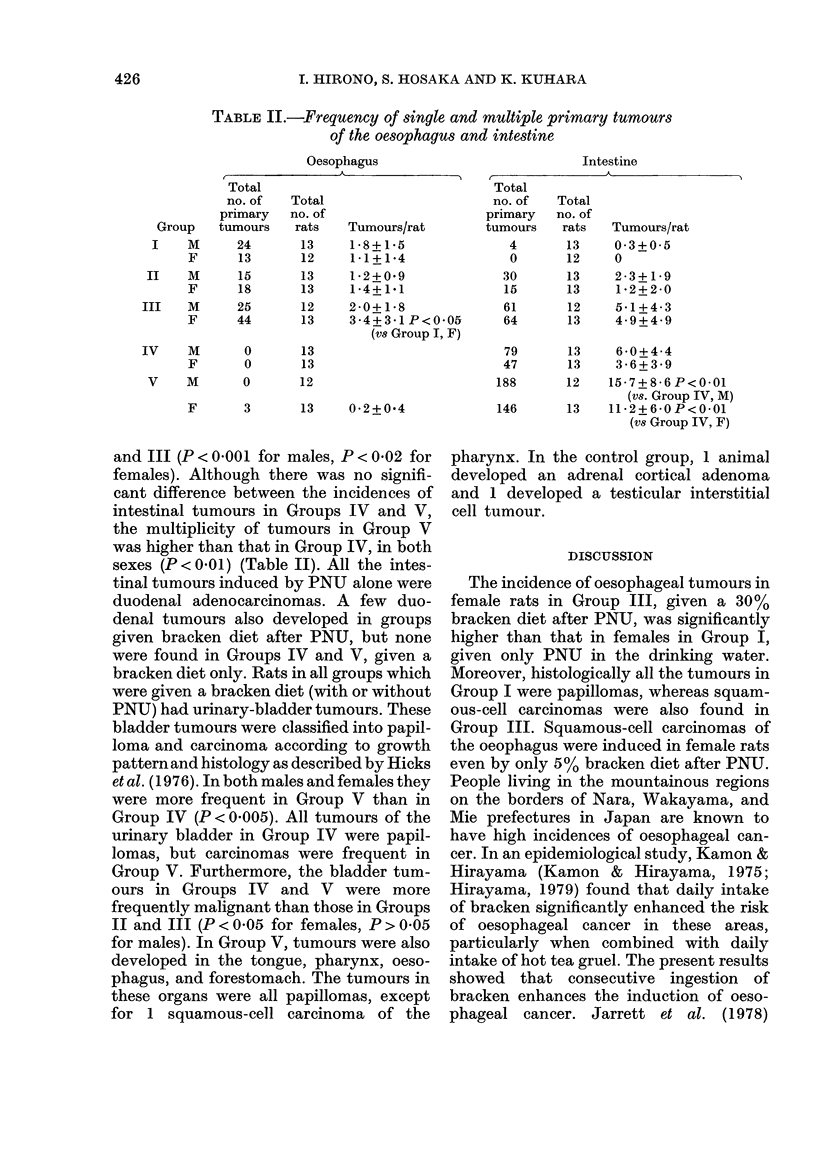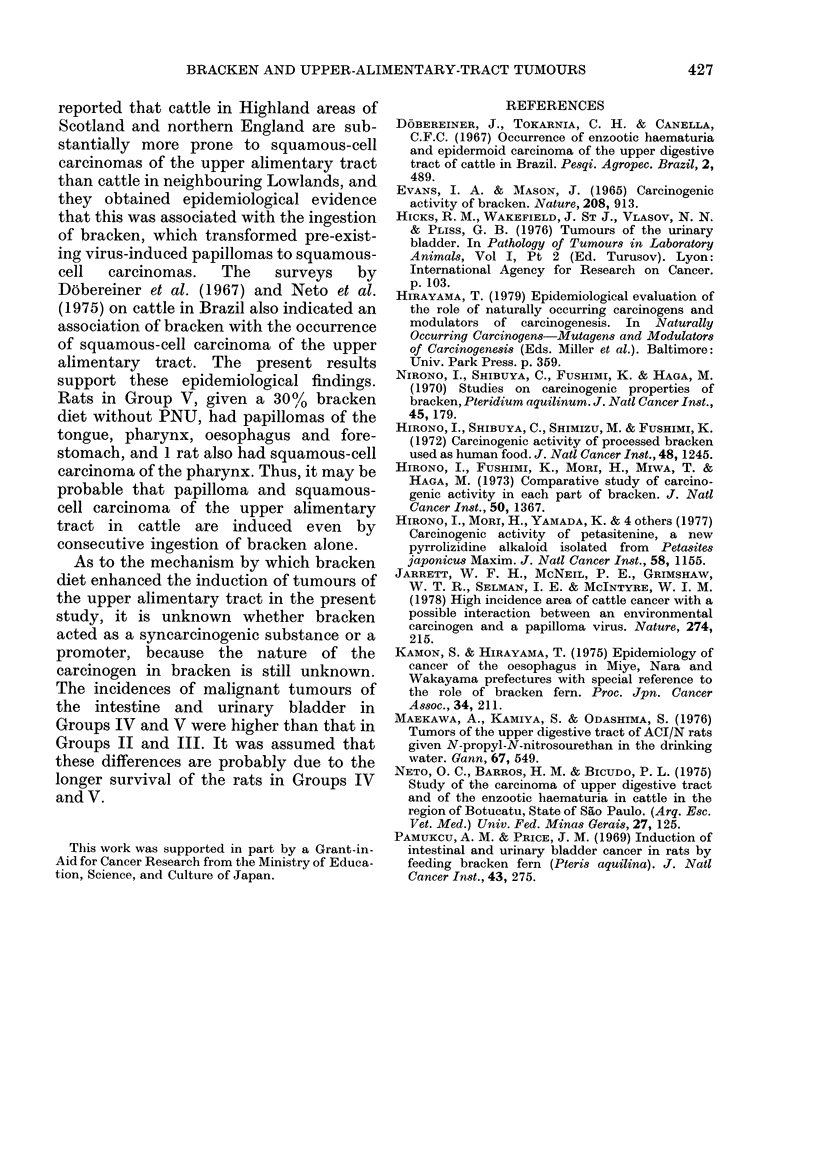# Enhancement by bracken of induction of tumours of the upper alimentary tract by N-propyl-N-nitrosourethan.

**DOI:** 10.1038/bjc.1982.219

**Published:** 1982-09

**Authors:** I. Hirono, S. Hosaka, K. Kuhara

## Abstract

The effect of bracken on the induction of tumours of the upper alimentary tract by N-propyl-N-nitrosourethan (PNU) was studied in 7-week-old ACI rats. Group I received a solution of 400 pts/10(6) of PNU in their drinking water for 6 weeks; Groups II and III were given PNU as in Group I, and then from 1 week later were fed on diets containing 5 and 30% bracken, respectively, for 33 weeks; Groups IV and V were fed on diets containing 5 and 30% bracken, respectively, for 33 weeks, from 14 weeks after birth. A control group was given basal diet and water only. The experiment was terminated after 40 weeks. The induction of tumours of the upper alimentary tract by PNU was enhanced by bracken diet; i.e. the incidence of pharyngeal tumours in male rats was significantly higher (P less than 0.025) in Group II (10/13) than in Group I (3/13). The incidence and multiplicity of oesophageal tumours in female rats were also higher in Group III than in Group I (P less than 0.025 for incidence; P less than 0.05 for multiplicity). Histologically, the oesophageal tumours in female rats in Groups II and III were not only papillomas but also squamous-cell carcinomas, whereas those in females of Group I were all papillomas. Furthermore, the incidence of tumours of the forestomach in female rats was also higher (P less than 0.05) in Group II (11/13) than in Group I (4/12).


*Br. J. Cancer* (1982) **46**, 423

### ENHANCEMENT BY BRACKEN OF INDUCTION OF TUMOURS OF THE UPPER ALIMENTARY TRACT BY *N*-PROPYL-*N*-NITROSOURETHAN

I. HIRONO, S. HOSAKA AND K. KUHARA

*From the Department of Carcinogenesis and Cancer Susceptibility, Institute of Medical Science, University of Tokyo, Shirokanedai* 4-6-1, *Minato-ku, Tokyo* 108, *Japan*

Received 6 October 1981 Accepted 7 April 1982

**Summary.—The effect of bracken on the induction of tumours of the upper alimentary tract by *N*-propyl-*N*-nitrosourethan (PNU) was studied in 7-week-old ACI rats. Group I received a solution of 400 pts/$10^6$ of PNU in their drinking water for 6 weeks; Groups II and III were given PNU as in Group I, and then from 1 week later were fed on diets containing 5 and 30% bracken, respectively, for 33 weeks; Groups IV and V were fed on diets containing 5 and 30% bracken, respectively, for 33 weeks, from 14 weeks after birth. A control group was given basal diet and water only. The experiment was terminated after 40 weeks.**

**The induction of tumours of the upper alimentary tract by PNU was enhanced by bracken diet; i.e. the incidence of pharyngeal tumours in male rats was significantly higher ($P < 0{\cdot}025$) in Group II (10/13) than in Group I (3/13). The incidence and multiplicity of oesophageal tumours in female rats were also higher in Group III than in Group I ($P < 0{\cdot}025$ for incidence; $P < 0{\cdot}05$ for multiplicity). Histologically, the oesophageal tumours in female rats in Groups II and III were not only papillomas but also squamous-cell carcinomas, whereas those in females of Group I were all papillomas. Furthermore, the incidence of tumours of the forestomach in female rats was also higher ($P < 0{\cdot}05$) in Group II (11/13) than in Group I (4/12).**

THE CARCINOGENICITY of bracken was most clearly demonstrated in the experiments of Evans & Mason (1965), who first found that multiple intestinal adenocarcinomas developed in rats fed bracken diet. Subsequently this was confirmed by Pamukcu & Price (1969) and by us (Hirono *et al.*, 1970, 1973). In Japan, young bracken fronds in the fiddlehead or crosier stage of growth are used as human food after their astringent taste has been removed with boiling water containing wood ash or sodium bicarbonate. This treatment has been shown greatly to reduce the carcinogenicity of bracken (Hirono *et al.*, 1972).

In Japan, the mountainous regions on the borders of Nara, Wakayama, and Mie prefectures are known to be endemic areas of cancer of the oesophagus. Kamon and Hirayama (1975) and Hirayama (1979) showed that daily intake of bracken significantly enhanced the risk of oesophageal cancer in these areas, particularly when combined with daily intake of hot tea gruel. Jarrett *et al.* (1978) reported a high incidence of squamous-cell carcinoma of the upper alimentary tract in cattle in Highland areas of Scotland and northern England. They presented evidence that this is associated with the presence of bracken, and that many carcinomas arise from pre-existing virus-induced papillomas, which occur in greater numbers in animals on the Highland farms than on Lowland farms. A high incidence of tumours of the upper alimentary tract in cattle was also found in regions where bracken grows in Brazil (Döbereiner *et al.*, 1967; Neto *et al.*, 1975). On the basis of these epidemiological findings, the role of bracken in induction of tumours of the upper alimentary tract was studied in rats treated with *N*-propyl-*N*-nitrosourethan

(PNU), which was previously shown to be carcinogenic by Maekawa *et al.* (1976).

### MATERIALS AND METHODS

*Animals and treatments.*—Inbred strain ACI rats 7 of weeks old were divided into 6 groups. The sexes and initial numbers of rats in each group are shown in Table I. Group I was given a solution of 400 pts/$10^6$ of PNU (Izumi Chemical Co., Inc., Yokohama, Japan) in distilled water to drink for 6 weeks. Groups II and III were also given a solution of PNU for 6 weeks, as in Group I, and then from 1 week later they were fed on diets containing 5 and 30% bracken, respectively, until the end of the experiment. Groups IV and V were given 5 and 30% bracken diets, respectively, from 14 weeks after birth. The control group was not given PNU or bracken. Rats were fed basal diet CE-2 (CLEA Japan Inc., Tokyo, Japan) except during periods on bracken diet. The composition of the basal diet was as described previously (Hirono *et al.*, 1977). Water was given *ad libitum* when PNU solution was not being given. The experiment was stopped after 40 weeks.

*Bracken.*—The bracken used in this study was collected in Hokkaido, in the northern part of Japan, in June. It was in the early stage of maturation, *i.e.* the tips of the fronds were still curled. The fresh bracken was dried at room temperature with a blower, ground and added in appropriate amounts to basal diet. All animals were necropsied when they died, or when they were killed either in a moribund condition during the experiment or at the end of the experiment. Tissues were fixed in 10% formalin, sectioned and stained with haematoxylin and eosin.

### RESULTS

The mean survival times of rats in each group are shown in Table I. Most rats in Group I, which received a PNU solution for 6 weeks, survived for more than 28 weeks, and 8 males and 11 females survived until the end of the experiment. Rats in Group II, given PNU and 5% bracken diet, showed a similar survival rate to those in Group I. Rats in Group III survived for significantly less time than those in Groups I ($P < 0{\cdot}01$ for males, $P < 0{\cdot}001$ for females) and II ($P < 0{\cdot}05$ for males, $P < 0{\cdot}01$ for females). In Group IV, given 5% bracken diet only, rats tended to survive for longer than those in Group II ($P < 0{\cdot}01$ for males, $P > 0{\cdot}05$ for females). A more significant difference in survival time was found between Groups V and III ($P < 0{\cdot}001$ for males, $P < 0{\cdot}01$ for females).

The incidences and histological types of tumours in each group are summarized in Table I. Tumours of the tongue developed in various incidences in all experimental groups. The incidence of pharyngeal tumours induced by PNU tended to be increased by bracken diet. This increase was most marked in male rats (10/13) in Group II, in which the incidence was significantly different ($P < 0{\cdot}025$) from that in males (3/13) in Group I. The incidence and multiplicity of oesophageal tumours in female rats induced by PNU (Group I) tended to be increased by the administration of bracken diet, especially in Group III ($P < 0{\cdot}025$ for incidence; $P < 0{\cdot}05$ for multiplicity, Table II).

Histologically, all the oesophageal tumours in female rats in Group I were papillomas, whereas squamous-cell carcinomas were also induced in most females in Groups II (7/13) and III (8/13) and one, in an animal in Group III, metastasized to the lymph nodes of the neck. The incidences of these squamous-cell carcinomas of the oeophagus in female rats were significantly higher in Group II ($P < 0{\cdot}02$) and Group III ($P < 0{\cdot}01$) than in Group I. The incidence of tumours of the forestomach induced by PNU was also increased by administration of bracken, and a significant difference was found between the incidences in female rats in Group I (4/12) and Group II (11/13) ($P < 0{\cdot}05$). Furthermore, although all tumours of the forestomach in females in Group I were papillomas, squamous-cell carcinomas were also induced in 5 females in Group II.

Rats in Groups IV and V, given bracken diet but not PNU, had a high incidence of jejunal and ileal tumours. Particularly, the incidence of adenocarcinoma in Groups IV and V was higher than that in Groups II

TABLE II.—*Frequency of single and multiple primary tumours of the oesophagus and intestine*

| Group | | Oesophagus: Total no. of primary tumours | Oesophagus: Total no. of rats | Oesophagus: Tumours/rat | Intestine: Total no. of primary tumours | Intestine: Total no. of rats | Intestine: Tumours/rat |
|---|---|---|---|---|---|---|---|
| I | M | 24 | 13 | $1 \cdot 8 \pm 1 \cdot 5$ | 4 | 13 | $0 \cdot 3 \pm 0 \cdot 5$ |
| | F | 13 | 12 | $1 \cdot 1 \pm 1 \cdot 4$ | 0 | 12 | 0 |
| II | M | 15 | 13 | $1 \cdot 2 \pm 0 \cdot 9$ | 30 | 13 | $2 \cdot 3 \pm 1 \cdot 9$ |
| | F | 18 | 13 | $1 \cdot 4 \pm 1 \cdot 1$ | 15 | 13 | $1 \cdot 2 \pm 2 \cdot 0$ |
| III | M | 25 | 12 | $2 \cdot 0 \pm 1 \cdot 8$ | 61 | 12 | $5 \cdot 1 \pm 4 \cdot 3$ |
| | F | 44 | 13 | $3 \cdot 4 \pm 3 \cdot 1$ $P < 0 \cdot 05$ (*vs* Group I, F) | 64 | 13 | $4 \cdot 9 \pm 4 \cdot 9$ |
| IV | M | 0 | 13 | | 79 | 13 | $6 \cdot 0 \pm 4 \cdot 4$ |
| | F | 0 | 13 | | 47 | 13 | $3 \cdot 6 \pm 3 \cdot 9$ |
| V | M | 0 | 12 | | 188 | 12 | $15 \cdot 7 \pm 8 \cdot 6$ $P < 0 \cdot 01$ (*vs.* Group IV, M) |
| | F | 3 | 13 | $0 \cdot 2 \pm 0 \cdot 4$ | 146 | 13 | $11 \cdot 2 \pm 6 \cdot 0$ $P < 0 \cdot 01$ (*vs* Group IV, F) |

and III ($P < 0{\cdot}001$ for males, $P < 0{\cdot}02$ for females). Although there was no significant difference between the incidences of intestinal tumours in Groups IV and V, the multiplicity of tumours in Group V was higher than that in Group IV, in both sexes ($P < 0{\cdot}01$) (Table II). All the intestinal tumours induced by PNU alone were duodenal adenocarcinomas. A few duodenal tumours also developed in groups given bracken diet after PNU, but none were found in Groups IV and V, given a bracken diet only. Rats in all groups which were given a bracken diet (with or without PNU) had urinary-bladder tumours. These bladder tumours were classified into papilloma and carcinoma according to growth pattern and histology as described by Hicks *et al.* (1976). In both males and females they were more frequent in Group V than in Group IV ($P < 0{\cdot}005$). All tumours of the urinary bladder in Group IV were papillomas, but carcinomas were frequent in Group V. Furthermore, the bladder tumours in Groups IV and V were more frequently malignant than those in Groups II and III ($P < 0{\cdot}05$ for females, $P > 0{\cdot}05$ for males). In Group V, tumours were also developed in the tongue, pharynx, oesophagus, and forestomach. The tumours in these organs were all papillomas, except for 1 squamous-cell carcinoma of the pharynx. In the control group, 1 animal developed an adrenal cortical adenoma and 1 developed a testicular interstitial cell tumour.

### DISCUSSION

The incidence of oesophageal tumours in female rats in Group III, given a 30% bracken diet after PNU, was significantly higher than that in females in Group I, given only PNU in the drinking water. Moreover, histologically all the tumours in Group I were papillomas, whereas squamous-cell carcinomas were also found in Group III. Squamous-cell carcinomas of the oeophagus were induced in female rats even by only 5% bracken diet after PNU. People living in the mountainous regions on the borders of Nara, Wakayama, and Mie prefectures in Japan are known to have high incidences of oesophageal cancer. In an epidemiological study, Kamon & Hirayama (Kamon & Hirayama, 1975; Hirayama, 1979) found that daily intake of bracken significantly enhanced the risk of oesophageal cancer in these areas, particularly when combined with daily intake of hot tea gruel. The present results showed that consecutive ingestion of bracken enhances the induction of oesophageal cancer. Jarrett *et al.* (1978)

reported that cattle in Highland areas of Scotland and northern England are substantially more prone to squamous-cell carcinomas of the upper alimentary tract than cattle in neighbouring Lowlands, and they obtained epidemiological evidence that this was associated with the ingestion of bracken, which transformed pre-existing virus-induced papillomas to squamous-cell carcinomas. The surveys by Döbereiner *et al.* (1967) and Neto *et al.* (1975) on cattle in Brazil also indicated an association of bracken with the occurrence of squamous-cell carcinoma of the upper alimentary tract. The present results support these epidemiological findings. Rats in Group V, given a 30% bracken diet without PNU, had papillomas of the tongue, pharynx, oesophagus and fore-stomach, and 1 rat also had squamous-cell carcinoma of the pharynx. Thus, it may be probable that papilloma and squamous-cell carcinoma of the upper alimentary tract in cattle are induced even by consecutive ingestion of bracken alone.

As to the mechanism by which bracken diet enhanced the induction of tumours of the upper alimentary tract in the present study, it is unknown whether bracken acted as a syncarcinogenic substance or a promoter, because the nature of the carcinogen in bracken is still unknown. The incidences of malignant tumours of the intestine and urinary bladder in Groups IV and V were higher than that in Groups II and III. It was assumed that these differences are probably due to the longer survival of the rats in Groups IV and V.

This work was supported in part by a Grant-in-Aid for Cancer Research from the Ministry of Education, Science, and Culture of Japan.

### REFERENCES

DÖBEREINER, J., TOKARNIA, C. H. & CANELLA, C.F.C. (1967) Occurrence of enzootic haematuria and epidermoid carcinoma of the upper digestive tract of cattle in Brazil. *Pesqi. Agropec. Brazil*, **2**, 489.

EVANS, I. A. & MASON, J. (1965) Carcinogenic activity of bracken. *Nature*, **208**, 913.

HICKS, R. M., WAKEFIELD, J. ST J., VLASOV, N. N. & PLISS, G. B. (1976) Tumours of the urinary bladder. In *Pathology of Tumours in Laboratory Animals*, Vol I, Pt 2 (Ed. Turusov). Lyon: International Agency for Research on Cancer. p. 103.

HIRAYAMA, T. (1979) Epidemiological evaluation of the role of naturally occurring carcinogens and modulators of carcinogenesis. In *Naturally Occurring Carcinogens—Mutagens and Modulators of Carcinogenesis* (Eds. Miller *et al.*). Baltimore: Univ. Park Press. p. 359.

NIRONO, I., SHIBUYA, C., FUSHIMI, K. & HAGA, M. (1970) Studies on carcinogenic properties of bracken, *Pteridium aquilinum*. *J. Natl Cancer Inst.*, **45**, 179.

HIRONO, I., SHIBUYA, C., SHIMIZU, M. & FUSHIMI, K. (1972) Carcinogenic activity of processed bracken used as human food. *J. Natl Cancer Inst.*, **48**, 1245.

HIRONO, I., FUSHIMI, K., MORI, H., MIWA, T. & HAGA, M. (1973) Comparative study of carcinogenic activity in each part of bracken. *J. Natl Cancer Inst.*, **50**, 1367.

HIRONO, I., MORI, H., YAMADA, K. & 4 others (1977) Carcinogenic activity of petasitenine, a new pyrrolizidine alkaloid isolated from *Petasites japonicus* Maxim. *J. Natl Cancer Inst.*, **58**, 1155.

JARRETT, W. F. H., MCNEIL, P. E., GRIMSHAW, W. T. R., SELMAN, I. E. & MCINTYRE, W. I. M. (1978) High incidence area of cattle cancer with a possible interaction between an environmental carcinogen and a papilloma virus. *Nature*, **274**, 215.

KAMON, S. & HIRAYAMA, T. (1975) Epidemiology of cancer of the oesophagus in Miye, Nara and Wakayama prefectures with special reference to the role of bracken fern. *Proc. Jpn. Cancer Assoc.*, **34**, 211.

MAEKAWA, A., KAMIYA, S. & ODASHIMA, S. (1976) Tumors of the upper digestive tract of ACI/N rats given *N*-propyl-*N*-nitrosourethan in the drinking water. *Gann*, **67**, 549.

NETO, O. C., BARROS, H. M. & BICUDO, P. L. (1975) Study of the carcinoma of upper digestive tract and of the enzootic haematuria in cattle in the region of Botucatu, State of São Paulo. (*Arq. Esc. Vet. Med.*) *Univ. Fed. Minas Gerais*, **27**, 125.

PAMUKCU, A. M. & PRICE, J. M. (1969) Induction of intestinal and urinary bladder cancer in rats by feeding bracken fern (*Pteris aquilina*). *J. Natl Cancer Inst.*, **43**, 275.